# Hierarchical Bayesian continuous‐time dynamic modeling approach to investigate the dynamic interplay between aerobic exercise and time‐locked cognitive performance in older adults with AD and metabolic risk

**DOI:** 10.1002/alz.085186

**Published:** 2025-01-09

**Authors:** Svenja Schwarck, Manuel C Voelkle, Jose Bernal, David Berron, Hannah Baumeister, Helena M. Gellersen, Marcel Daamen, Henning Boecker, Gabriel Ziegler, Emrah Düzel

**Affiliations:** ^1^ German Center for Neurodegenerative Diseases (DZNE), Magdeburg Germany; ^2^ Institute of Cognitive Neurology and Dementia Research (IKND), Otto‐von‐Guericke University, Magdeburg Germany; ^3^ Humboldt University of Berlin, Berlin, Berlin Germany; ^4^ German Center for Neurodegenerative Diseases (DZNE), Bonn Germany

## Abstract

**Background:**

Training studies report beneficial effects of physical (PP) on cognitive performance (COG) in older adults, but are often accompanied by potentially biased parameters, conclusions, and lack of directionality. To address these issues, we used a dynamic Bayesian approach to analyse the dynamic session‐to‐session change and coupling of PP and COG over time.

**Methods:**

We used two studies (N = 17 each): Study 1 contained 24‐weeks (72 sessions) of training of older adults with suspected Alzheimer’s disease (AD). Study 2 included four months (40 sessions) of training of older adults at metabolic risk. The hierarchical Bayesian continuous‐time dynamic modeling approach (R package ctsem) comprised: (i) A subject‐level latent dynamic model, (ii) subject‐level measurement model, and (iii) population model. The dynamic model was specified with two fully connected state variables enabling bidirectional coupling between PP and COG using default priors and starting values (4 chains and 8.000 iterations). Intercept and drift parameters were set to vary freely. Population and individual level parameters are estimated simultaneously using all data from all subjects. Second‐level models included MMSE, SF12 (physical health questionnaire) in study 1 and whole hippocampal volumes (wHCV: T2‐ASHS, longitudinal) and white matter hyperintensities (SAMSEG, longitudinal) in study 2 as time independent covariates.

**Results:**

Study 1: Higher PP was dynamically linked to COG (‐1.335, 95%‐BCI [‐1.725, ‐0.954]). The effect was short‐term, lasting up to five days (‐0.368, 95%‐BCI [‐0.479, ‐0.266]). Study 2: PP improved COG in subsequent sessions (‐1.11, 95%‐BCI[‐1.22, ‐0.99]), which lasted for up to 15 days. Higher wHCV was associated with a stronger coupling‐effect of PP on COG (‐0.15, 95%‐BCI[‐0.18, ‐0.11] ) and lower persistence of COG (‐0.145, 95%‐BCI[‐0.21, ‐0.08]).

**Discussion:**

Our results show immediate exercise‐induced improvements in COG, with a longer‐lasting effect for participants at metabolic risk compared to older adults with suspected AD. Despite impaired cognitive performance, the cognitive system was still able to fluctuate and change favorably. Physical exercise seems to mobilize capacities that would otherwise remain unused. Utilizing a Bayesian approach to examine time‐locked effects can provide insights for implementing individualized training approaches and guiding clinical decision‐making.

